# Novel Tertiary Amino Containing Blinding Composite Membranes via Raft Polymerization and Their Preliminary CO_2_ Permeation Performance

**DOI:** 10.3390/ijms16059078

**Published:** 2015-04-23

**Authors:** Lifang Zhu, Mali Zhou, Shanshan Yang, Jiangnan Shen

**Affiliations:** 1 School of Geomatics and Municipal Engineering, Zhejiang University of Water Resources and Electric Power, Hangzhou 310014, China; E-Mail: zhulifang1010@zju.edu.cn; 2 Ocean College, Zhejiang University of Technology, Hangzhou 310014, China; E-Mails: maryzjut@163.com (M.Z.); yangss97@163.com (S.Y.)

**Keywords:** reversible addition fragmentation chain transfer (RAFT) polymerization, divinyl benzene, star polymer, CO_2_ facilitated carrier

## Abstract

Facile synthesis of poly (*N*,*N*-dimethylaminoethyl methacrylate) (PDMAEMA) star polymers on the basis of the prepolymer chains, PDMAEMA as the macro chain transfer agent and divinyl benzene (DVB) as the cross-linking reagent by reversible addition-fragmentation chain transfer (RAFT) polymerization was described. The RAFT polymerizations of DMAEMA at 70 °C using four RAFT agents with different R and Z group were investigated. The RAFT agents used in these polymerizations were dibenzyl trithiocarbonate (DBTTC), s-1-dodecyl-s'-(α,α'-dimethyl-α-acetic acid) trithiocarbonate (MTTCD), s,s'-bis (2-hydroxyethyl-2'-dimethylacrylate) trithiocarbonate (BDATC) and s-(2-cyanoprop-2-yl)-s-dodecyltrithiocarbonate (CPTCD). The results indicated that the structure of the end-group of RAFT agents had significant effects on the ability to control polymerization. Compared with the above-mentioned RAFT agents, CPTCD provides better control over the molecular weight and molecular weight distribution. The polydispersity index (PDI) was determined to be within the scope of 1.26 to 1.36. The yields, molecular weight, and distribution of the star polymers can be tuned by changing the molar ratio of DVB/PDMAEMA-CPTCD. The chemical composition and structure of the linear and star polymers were characterized by GPC, FTIR, ^1^H NMR, XRD analysis. For the pure Chitosan membrane, a great improvement was observed for both CO_2_ permeation rate and ideal selectivity of the blending composite membrane upon increasing the content of SPDMAEMA-8. At a feed gas pressure of 37.5 cmHg and 30 °C, the blinding composite membrane (Cs: SPDMAEMA-8 = 4:4) has a CO_2_ permeation rate of 8.54 × 10^−4^ cm^3^ (STP) cm^−2^∙s^−1^∙cm∙Hg^−1^ and a N_2_ permeation rate of 6.76 × 10^−5^ cm^3^ (STP) cm^−2^∙s^−1^∙cm∙Hg^−1^, and an ideal CO_2_/N_2_ selectivity of 35.2.

## 1. Introduction

Fixed carrier membranes containing the CO_2_ facilitated carriers (*i.e.*, amino and carboxyl groups) have both high selectivity and high permeability compared with conventional polymeric membranes, which is very attractive [1,2,3]. The facilitated transport in which carbon dioxide reversibly reacts with a carrier is a solution for conventional membranes that considerably enhances the CO_2_ permeability. Recently, much effort has gone into the development of the membrane separation technique and it is mainly manifested in two aspects: new membrane materials selection [4,5,6,7,8] and preparation of the membrane materials [9,10,11,12]. For gas permeation, the formation of crystals in polymer membranes due to the strong intermolecular interaction is generally deleterious since they act as impermeable obstacles to gas molecular transport [13]. It has been found that the polyvinylamine membrane showed lower CO_2_/CH_4_ permselectivity in higher carrier content due to high crystallinity of polyvinylamine reducing effective permeating areas and effective carriers. Dendrimer might be a solution to the problem [14]. Starburst dendrimer has a large amount of terminal groups, high branched structure, and non crystallinity, which make it a potential material for gas separation membrane. Sirkar firstly reported CO_2_ separation membrane using a dendrimer as membrane material, the membrane exhibited excellent separation performance [15]. Kazama developed a composite membrane of polyamidoamine dendrimer with a chitosan modified substract [16]. Recenly, Wang fabricated a composite membrane using pentaerythriyl tetraethylenediamine dendrimer as membrane material [8].

The star polymer is similar to a dendrimer, it consists of multiple linear chains linked together at one end of each chain by junction points, and has attracted considerable attention in recent years due to smaller hydrodynamic dimensions, higher degree of functionalities, lower degree of crystallinities and viscosities compared with the linear counterparts of the same molecular weight [17,18,19]. So those polymers have a potential application on membrane separation technique [20,21]. Zhao *et al.* [22] have successfully synthesized novel porous membranes by blending amphiphilic hyperbranched-star polymers (HPE-g-MPEG) with PVDF via the phase inversion process. The blend membranes with longer arm HPE-g-MPEGs show lower static protein adsorption, higher protein solution fluxes, and better protein solution flux recovery than the pure PVDF membrane. Wodzki [23] reported the application of some star-shaped polymers (SSP) for transport and separation of metal cations in a liquid membrane system. Suda *et al.* [24] prepared sulfonated star-hyperbranched polyimide membranes and measured their proton conductivities. The results show that a good proton-transport pathway in the core-shell structure was formed when star-hyperbranched polyimide membranes with high molecular weight and this proton exchange membrane might have potential applications in fuel cells. But to our knowledge, there are few reports on application of star polymers on facilitated transport membranes for selectively removing CO_2_ from gas mixtures. Star PDMAEMA contains tertiary amine groups, which might act as carriers for CO_2_ facilitated transport.

One of the major challenges in well-defined star polymer synthesis is the control of molecular weight with narrow polydispersity index (PDI) [25], although preparation of star polymers has been documented as early as 1948 by Schaefgen and Florey [26]. Well-defined star polymers are mainly prepared by living anionic polymerization [27,28], cationic polymerization [29], and “living”/controlled radical polymerization [30,31]. In 1956, Morton and his coworkers [32] were able to take advantage of the living anionic polymerization to synthesize well-defined four-armed polystyrenes for the first time by neutralizing living poly (styryllithium) with tetrachlorosilane. “Living”/controlled radical polymerization (LCRP) approaches such as iniferter-mediated polymerization [33], nitroxide-mediated free-radical polymerization (NMRP) [34], atom transfer radical polymerization (ATRP) [35,36], and reversible addition-fragmentation chain transfer (RAFT) polymerization [37,38], have been efficiently used to synthesize a variety of star polymers with controlled composition and molecular weight. Among these methods, RAFT polymerization is a facile and versatile method to prepare star polymers because of its many advantages such as relatively mild reaction conditions, wide range of monomers, tolerance of various functional groups, and lack of metal catalyst.

Regardless of the polymerization methods (ionic or radical), preparations of star polymers described in the literature can be categorized into two broad approaches, namely, (i) the arm first [39,40,41] and (ii) the core first approaches [42,43]. The arm first approach involves the synthesis of prefabricated arms, usually through “living”/controlled polymerization, followed by coupling reaction of living polymer chains with a cross-linking reagent (e.g., divinylbenzene or tetrachlorosilane). The core first approach involves the use of multifunctional initiator (the core) followed by the extension of arms through polymerization. In comparison with the core first approach, arm first approach can produce the stars with a larger number of arms. Thus the arm first method has been extensively utilized in the synthesis of various star polymers via “living”/controlled radical polymerization.

Although star polymers have unique properties compared to their linear analogues, they have small intermolecular forces and lack a significant entanglement in the solid state, indicating that it is very difficult to prepare tough membranes with a good mechanical strength [24]. Therefore, in order to improve the mechanical properties, we developed a novel facilitated transport membrane by blending chitin (Cs), a good material for preparing membranes, with star PDMAEMA via RAFT polymerization, and investigated its gas permeation properties by using humidified feed gas and sweep gas because star PDMAEMA only contains a tertiary amine group as the CO_2_ facilitated carrier.

## 2. Results and Discussion

### 2.1. Effect of Various Experimental Parameters on RAFT Polymerization of DMAEMA

#### 2.1.1. Polymerization of DMAEMA Using Dithio and Trithio Compounds as a Source of CTA

**Figure 1:**
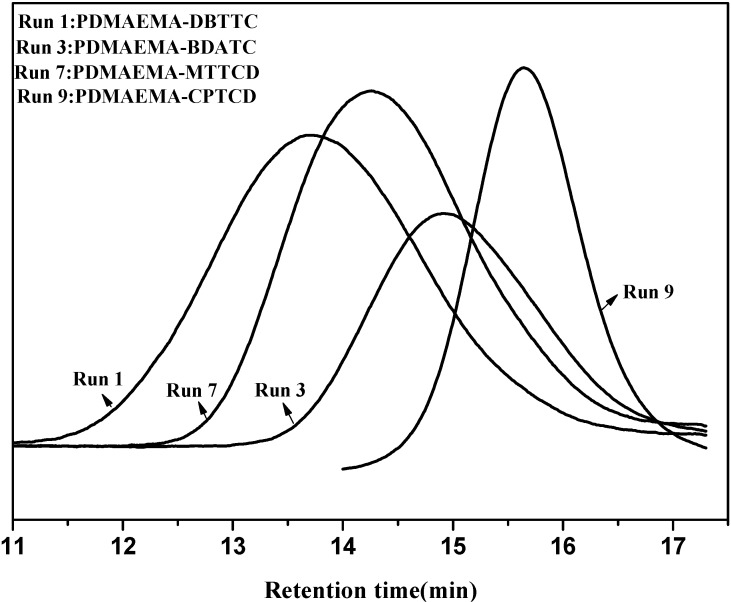
The gel permeation chromatograph (GPC) of poly (*N*,*N*-dimethylaminoethyl methacrylate) (PDMAEMA) with different reversible addition-fragmentation chain transfer (RAFT) agents(DMAEMA:DBTTC/BDATC/MTTCD/CPTCD:AIBN = 100:1:0.25, reaction time = 6 h).

A series of RAFT polymerizations of DMAEMA were performed under different conditions to investigate the influence of several experimental parameters such as CTA, CTA/initiator molar ratio, monomer/CTA molar ratio, and reaction time on the controlled character of the process. The RAFT polymerizations of DMAEMA with DBTTC, BDATC, MTTCD and CPTCD as RAFT agents and AIBN as initiator, respectively, were carried out. The conditions and results are summarized in Table 1. The GPC curves of the obtained prepolymers corresponding to Run 1, Run 3, Run 7, Run 9 were shown in Figure 1.

**Table 1:** RAFT polymerization results of DMAEMA in the presence of DBTTC, MTTCD, BDATC and CPTCD.

Run	CTA	Time (h)	Monomer/RAFT/Initiator (M/R/I)	Conversion ^c^ (%)	*M*_n_^a^ (GPC)	*M*_n_^b^ (Theoretical)	*M*_W_/*M*_n_ (PDI)
1	DBTTC	6	100:1:0.25	55.4	42,218	8998	2.20
2	DBTTC	6	100:1:0.33	61.4	36,090	9903	2.41
3	BDATC	6	100:1:0.25	31.3	15,894	5201	1.37
4	BDATC	6	100:1:0.33	43.0	12,783	7032	1.42
5	BDATC	6	200:1:0.25	20.8	42,472	6880	1.54
6	BDATC	10	100:1:0.25	43.0	19,001	7308	1.57
7	MTTCD	6	100:1:0.25	52.1	21,415	8544	1.81
8	CPTCD	6	60:1:0.25	30.6	5230	3225	1.26
9	CPTCD	6	100:1:0.25	45.5	8325	7484	1.27
10	CPTCD	6	100:1:0.33	45.5	7879	7491	1.29
11	CPTCD	6	200:1:0.25	25.17	9570	8250	1.32
12	CPTCD	6	300:1:0.25	35.7	18,833	17,160	1.36
13	CPTCD	12	100:1:0.25	47.1	8132	7740	1.30

^a^ *M*_n_ value obtained by GPC; ^b^ computed by equation: *M*_n_(th) = *M*_RAFT_ + n·Conv·*M*_DMAEMA_; ^c^ Conversion calculated based on the gravimetric method. Reaction temperature 70 °C, V_toluene_ = 14.0 mL.

In the RAFT process, one of the key factors for synthesizing well-defined polymers is the design of CTA, as the choice of the Z and R groups depends on the monomer [44]. When DMAEMA was polymerized in the presence of DBTTC, MTTCD and BDATC (Run 1–2, Run 7 and Run 3–6), the resultant PDMAEMA has a polydispersity index (PDI) >1.50 and 1.37~1.57, respectively. The reason for this phenomenon is probably that the balance of the reversible addition-fragmentation chain transfer is disturbed by an unapt match of monomer and CTA. However, in the presence of CPTCD (run 8–13), the polymerization showed a better control over the relative molecular mass and distribution. The polymer obtained was narrow in polydispersity index (<1.36) and the theoretical number average molecular weight *M*_n,th_ was in good agreement with *M*_n,GPC_. Run 9 and Run 13 were performed under same conditions except reaction time. When the reaction time of Run 13 was doubled, neither the molecular weight of polymer nor the conversion of DMAEMA were improved. This may be because the living chains had lost activity and become dead chains. The polymerization is controlled at low ratios of DMAEMA:CTA, while at higher ratios, the polymerization is plagued by transferring to solvent. The contribution of transferring to solvent could have been attenuated by polymerizing at higher monomer concentration, but at such concentration, the increase of viscosity will result in a loss of control and broad PDIs [45]. It is apparent that, throughout the reaction, the experimental *M*_n_ values are higher than the theoretical values calculated by Equation (1). There may be two reasons that lead to this phenomenon. One reason is that the first transferring between CTA and a propagating radical is less efficient than the subsequent transferring between a dormant chain and a propagating radical [46]. The other is that, in this study the measured *M*_n_ values are relative to narrow molecular weight distribution polymethyl methacrylate (PMMA) and the discrepancy may be attributed to the inadequacy of PMMA standards to approximate the hydrodynamic volume of PDMAEMA in DMF + 0.05M LiBr, a calibration phenomenon [47]. Combining Table 1 with Figure 1, we can see that the polymerization could be controlled by changing RAFT agent or that the molar ratio and CPTCD was the most effective RAFT agent for well-controlled polymerization of DMAEMA.

#### 2.1.2. Kinetics of DMAEMA Polymerization in the Presence of CPTCD

**Figure 2:**
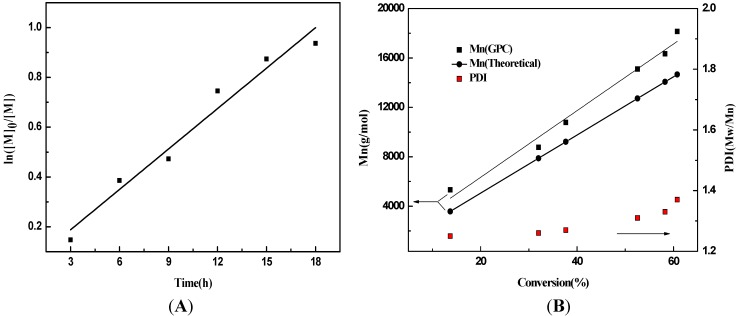
(**A**) Dependence of ln ([M]_0_/[M]) on time (h); (**B**) Relationship of the molecular weights and polydispersity index (PDI) of PDMAEMA macro-chain transfer agent obtained with monomer conversion using AIBN as the radical initiator and CPTCD as the RAFT agent in toluene at 70 °C. DMAEMA/CPTCD/AIBN = 150:1:0.25, C_DMAEMA,0_ = 2.0 mol/L.

The performance of the radical polymerization with CPTCD as RAFT agent was explored. DMAEMA was polymerized at 70 °C with AIBN as the initiator, and the feed molar ratio of reactants is DMAEMA:CPTCD:AIBN = 150:1:0.25. The time-conversion and the pseudo first-order kinetic plots are shown in Figure 2A. The linearity of the pseudo first-order kinetic plot suggests that there is a constant radical concentration throughout the RAFT polymerization and the polymerization occurs in a controlled manner. Linear growth of the molecular weight with the increase of monomer conversion was shown in Figure 2B. The PDI increases slightly with the conversion but remains in the range of 1.25~1.37. These results confirm that the polymerization of DMAEMA by the RAFT process in the presence of CPTCD follows a virtually “living” polymerization mechanism.

### 2.2. Star Polymers with PDMAEMA Arms

#### 2.2.1. Influence of DVB/PDMAEMA-CPTCD Ratio

**Figure 3:**
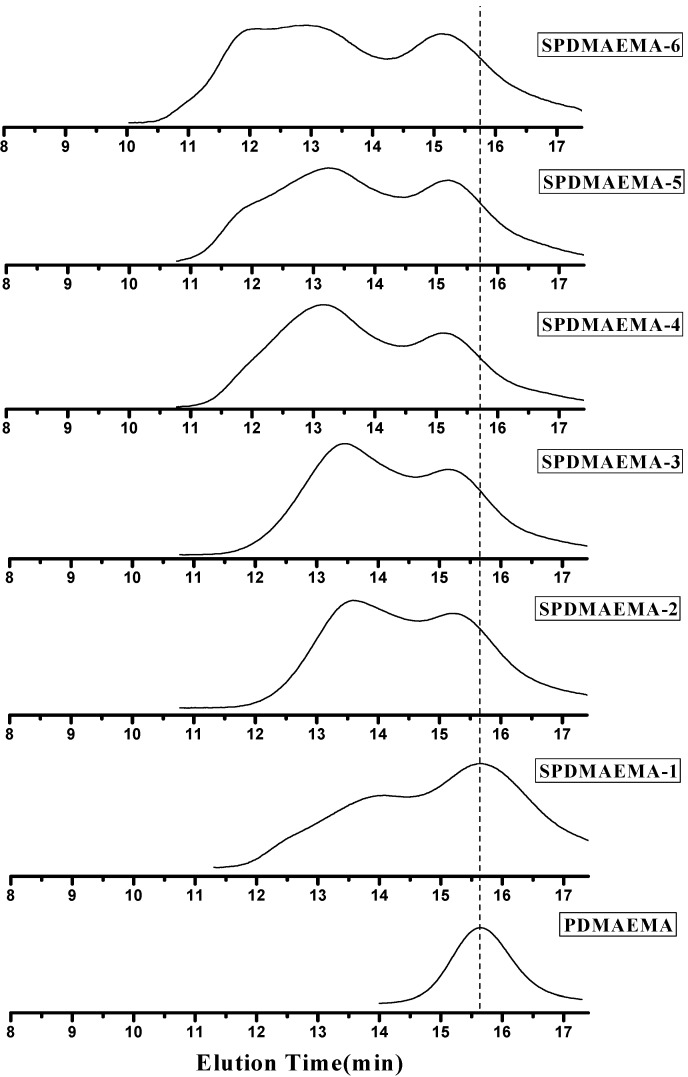
Gel permeation chromatograph (GPC) traces of SPDMAEMA with the DVB/PDMAEMA-CPTCD = 25 (SPDMAEMA-1); 35 (SPDMAEMA-2); 40 (SPDMAEMA-3); 45(SPDMAEMA-4); 50 (SPDMAEMA-5); 55 (SPDMAEMA-6) obtained for 12 h polymerization at 100 °C in the presence of AIBN as initiator (molar ratio: PDMAEMA-CPTCD/AIBN = 1:0.5), PDMAEMA-CPTCD: 0.4150 g, Toluene: 8.26 mL.

There have been a lot of research reports about star polymer preparation via arm-first method. For instance, Pan* et al.* [17] applied the linear macro RAFT agent to prepare star polymer via arm-first method. It was found that the formation of star polymer by arm-first method was affected by molecular weight of macro-CTA or macro-initiator, feed ratios and polymerization time in the previous literature [48]. In this work, in order to convert almost all of the linear polymer chains to the star polymers, the polymerization time on the RAFT polymerization of DVB was set to be 12 h. The effects of the ratio of DVB to PDMAEMA-CPTCD (*M*_n,GPC_ = 8325 g/mol, PDI = 1.27) were studied, and the results were listed in Table 2. The corresponding GPC traces were shown in Figure 3. In general, the molecular weight of polymer stars increased with the increasing molar ratio of DVB to PDMAEMA-CPTCD. With the increase of DVB to PDMAEMA-CPTCD ratio, the first peak at lower molecular weight position belonging to the linear macro RAFT agent, PDMAEMA-CPTCD, stayed at the same elution time and its integral area decreased gradually. Then, the second peak at higher molecular weight position increased continuously, which suggests that the polymerization of DVB occurred and more and more linear polymers changed to star polymers. When DVB:PDMAEMA-CPTCD = 50:1, a small third peak appeared, which might be owing to star-star coupling reaction and too high a ratio will lead to the gel effect (e.g., DVB:PDMAEMA-CPTCD = 60:1). The linear macro RAFT agent, which was not attached to PDMAEMA stars, still remained throughout the polymerization process.

**Table 2:** The effects of DVB/PDMAEMA-CPTCD molar ration on the formation of star polymers from RAFT polymerization of DVB ^a^.

Run	Sample	DVB/PDMAEMA Molar Ration	*M*_n_ (GPC) ^b^	*M*_W_ (GPC) ^b^	PDI
1	SPDMAEMA-1	25	46,725	78,498	1.68
2	SPDMAEMA-2	35	55,448	86,611	1.56
3	SPDMAEMA-3	40	64,458	109,048	1.69
4	SPDMAEMA-4	45	91,150	191,446	2.10
5	SPDMAEMA-5	50	96,692	230,403	2.38
6	SPDMAEMA-6	55	117,354	322,242	2.75
7 ^c^	SPDMAEMA-7	60	-	-	-
8 ^d^	SPDMAEMA-8	45	114,356	163,529	1.43

^a^ The polymerizations were carried out at 100 °C, reaction time is 12 h; initiator: AIBN (AIBN/PDMAEMA-CPTCD = 0.5/1), PDMAEMA-CPTCD (*M*_n,GPC_ = 8325 g/mol, PDI = 1.27): 0.415 g, Toluene: 8.26 mL; ^b^ Measured by GPC method; ^c^ Cross-linking reactions occurred during the polymerization; ^d^ The sample SPDMAEMA-8 was obtained by precipitation fractionation from SPDMAEMA-4.

#### 2.2.2. Separation of Star Polymers from Linear Polymer Contaminant

**Figure 4:**
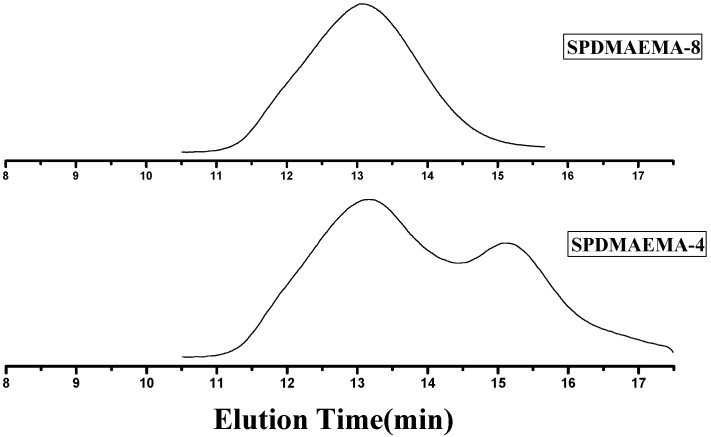
GPC traces of SPDMAEMA-4 and SPDMAEMA-8 (SPDMAEMA-8 was obtained by precipitation fractionation from SPDMAEMA-4).

From the GPC traces in Figure 3, we observed that the linear polymer decreased slowly throughout the RAFT process and even at high ratio, there was still some linear polymers left, which was due to the RAFT mechanism. During the RAFT process of DVB polymerization, there is a balance between the reversible addition and fragmentation reactions. The propagation reactions occurred both in the star polymers and DVB solution for the formation of PDMAEMA stars. Any linear polymer chain radical with trithiocarbonate group will produced a propagating radical and a CPTCD-terminated polymer linear chain. Therefore, the linear macro RAFT agent, PDMAEMA-CPTCD, was consumed slowly and it was difficult to get pure SPDMAEMA without linear polymers through RAFT method. In order to get pure star PDMAEMA, the precipitation fractionation technique was carried out to free of linear polymers contaminant from the star-shaped polymers. The petroleum ether was added slowly into the solution of SPDMAEMA-4 (Run 4 in Table 2) in THF (1%) until a small amount of turbidity was precipitated. The precipitation fractionation was repeated three times and SPDMAEMA-8 (Run 8 in Table 2, 32.90% yield) collected by filtration. Its GPC curve is shown in Figure 4.

### 2.3. Characterization

2.3.1. ^1^H NMR Analysis

**Figure 5:**
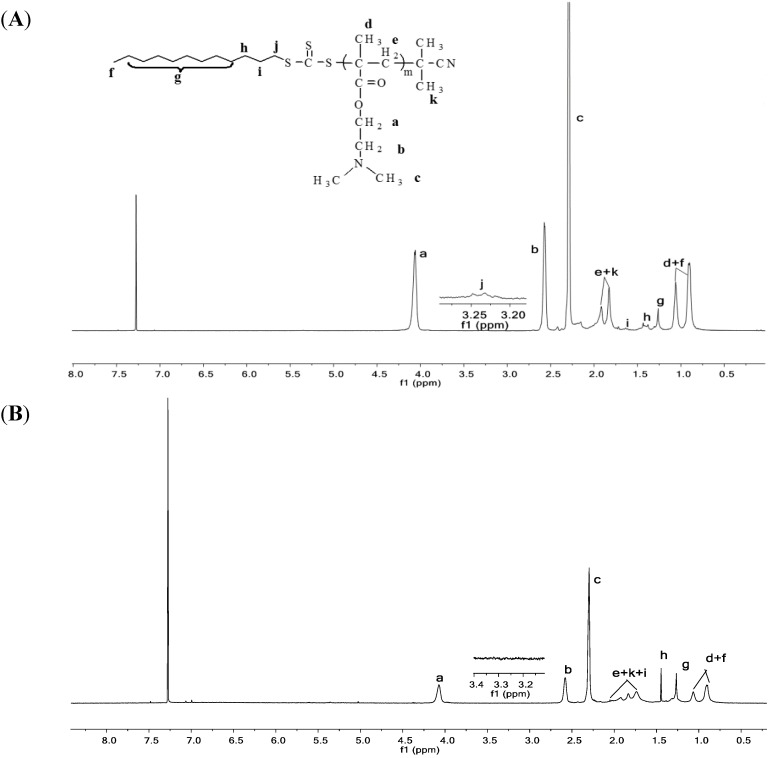
^1^H NMR spectra of PDMAEMA-CPTCD (**A**) and SPDMAEMA-8 (**B**).

The ^1^H NMR spectra of PDMAEMA-CPTCD and SPDMAEMA-8 was shown in Figure 5A,B. As shown in Figure 5A, the chemical shifts at 2.58 ppm (a) and 4.06 ppm (b) are assigned, respectively, to the methylene protons (–NCH_2_–) and (–OCH_2_–) of the PDMAEMA side chain. Besides the characteristic signals of PDMAEMA, the signal at 3.21~3.26 ppm (j) ascribes to the methylene protons next to the trithiocarbonate group and other signals arise from the methyl protons and the methylene protons of CPTCD. All these results indicate the linear macro RAFT agent, PDMAEMA-CPTCD, has been successfully synthesized. The signal at 7.28 ppm is attributed to solvent peak. ^1^H NMR spectrum of SPDMAEMA-8 was shown in Figure 5B. In comparison with Figure 5A, we could not see the proton signal at 3.21~3.26 ppm (j) ascribed to the methylene protons next to the trithiocarbonate group of CPTCD. Meanwhile, almost no characteristic signals ascribed to aromatic protons of DVB could be observed. The reason for this phenomenon may be that the PDMAEMA stars have tightly cross-linked cores, which is why we call these polymers star polymers [37,49].

#### 2.3.2. IR Analysis

**Figure 6:**
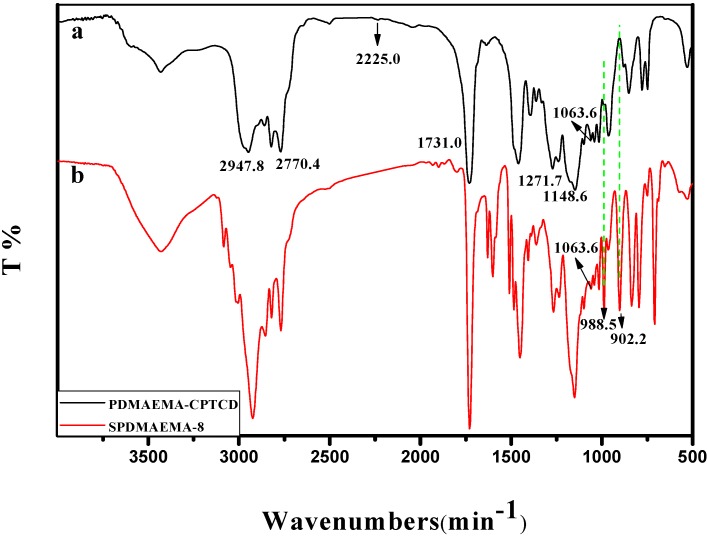
IR spectra of PDMAEMA-CPTCD (a) and SPDMAEMA-8 (b).

Figure 6 showed the FTIR spectra of PDMAEMA-CPTCD and SPDMAEMA-8. As illustrated in Figure 6a, the FTIR spectrum of PDMAEMA-CPTCD contains the characteristic band for C=O stretching (*v* = 1731.0 cm^−1^), C-O stretching (*v* = 1271.7 cm^−1^), C–N stretching (*v* = 1148.6 cm^−1^) and C=S stretching (*v* = 1063.6 cm^−1^). Moreover, there are three strong absorption peaks around 2947.8 cm^−1^ attributed to the stretching vibrations of C–CH_3_ and –CH_2_– groups. The result of FTIR indicated that PDMAEMA-CPTCD was obtained and could be used as the linear macro RAFT agent. Compared to Figure 5A, we observed the out of plane bending vibration bands of C–H in carbon-carbon double bond of DVB unit in center of stars at *ν* = 902.2 and 989 cm^−1^, and the stretching vibration band of C=S at *ν* = 1063.6 cm^−1^ in Figure 6b. These facts further confirm the formation of the PDMAEMA stars with tightly cross-linked core.

#### 2.3.3. XRD Analysis

**Figure 7:**
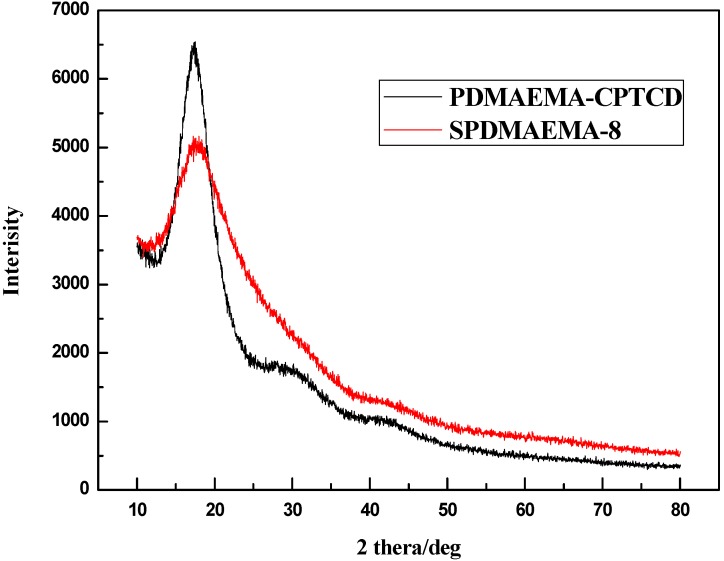
The XRD traces of PDMAEMA-CPTCD and SPDMAEMA-8.

XRD traces of PDMAEMA-CPTCD and SPDMAEMA-8 were shown in Figure 7. The diffraction peaks of PDMAEMA-CPTCD and SPDMAEMA-8 are formed by the amorphous diffuse peak, suggesting that they are completely amorphous polymers.

### 2.4. CO_2_ Separation Performance of the Blending Composite Membranes with Different Cs/SPDMAEMA-8 Volume Ratios under the Wet Condition

**Figure 8:**
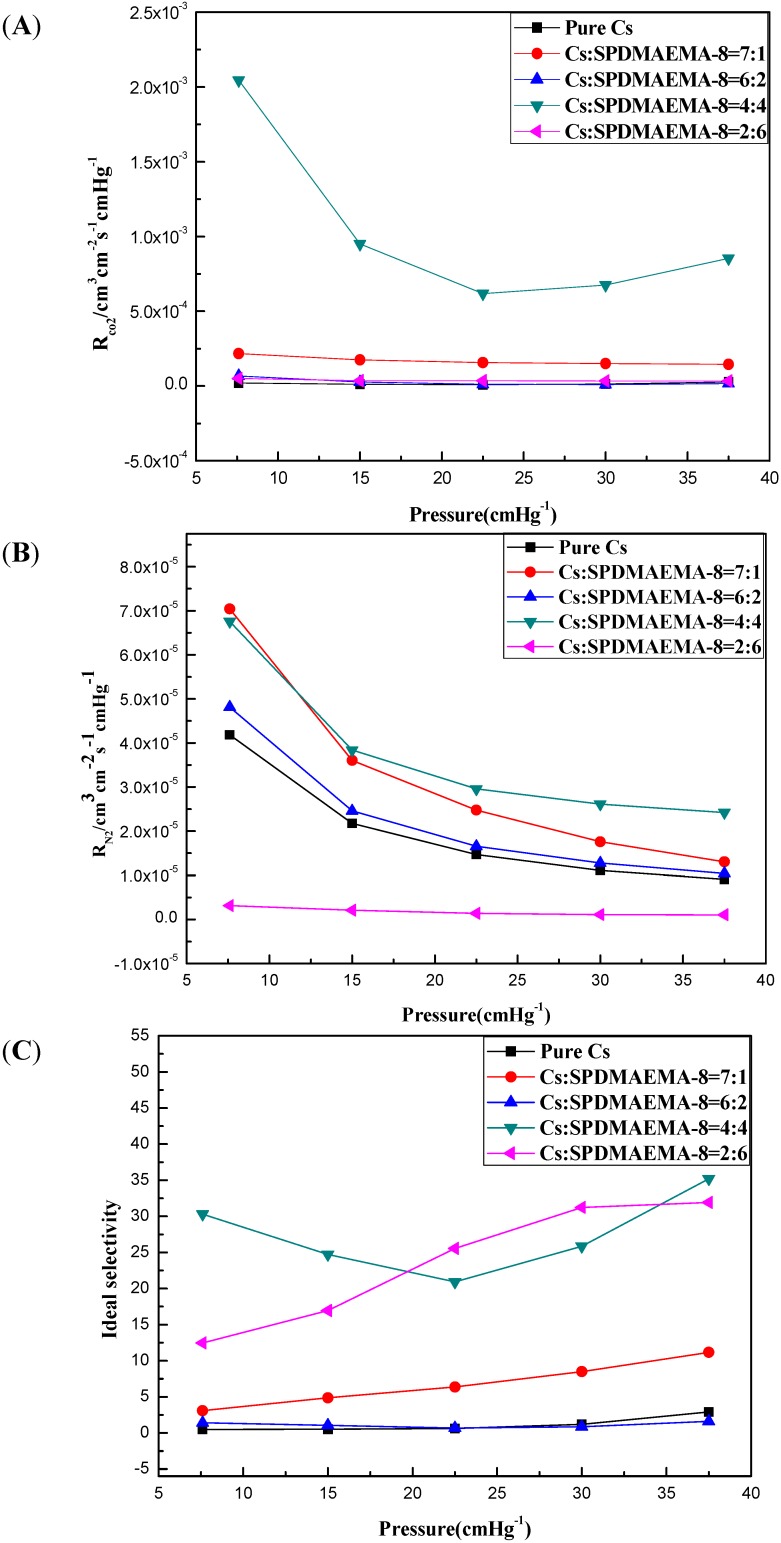
The permeability of the blinding composite membranes at different Cs/SPDMAEMA-8 volume ratios (**A**) CO_2_ permeation rate (**B**) N_2_ permeation rate (**C**) Ideal Selectivity of CO_2_ over N_2_ (Pure humidified gas, 30 °C).

Figure 8 showed the performance of the blending composite membranes with different Cs/SPDMAEMA-8 volume ratios (Cs:SPDMAEMA-8 = 8:0, 7:1, :6:2, 4:4, 2:6, 1:7) in different pressure: (a) CO_2_ permeation rate; (b) N_2_ permeation rate; (c) ideal selectivity of CO_2_ over N_2_. As illustrated in Figure 8, all blending composite membranes with different Cs/SPDMAEMA-8 volume ratios have the same trend as the feed gas pressure changes. With increasing feed gas pressure, CO_2_ permeation rate drops rapidly at a relatively low pressure (7.5–22.5 cmHg) and then decreases gently at a higher pressure (22.5–37.5 cmHg). This is due to the increscent pressure increasing the concentration of CO_2_ in the membrane, which results in facilitated transport reversible reaction the impact of the control. In comparison with CO_2_, the N_2_ permeation rate decreases slightly and does not change obviously when the feed gas pressure changes. This is because N_2_ follows the dissolution diffusion mechanism.

For the pure Cs membrane (2.0 wt % Cs casting membrane solution), CO_2_ permeation rate and ideal selectivity of the blending composite membrane both have a great improvement, but the N_2_ permeation rate does not significantly change when increasing the content of SPDMAEMA-8. For instance, at a feed gas pressure of 37.5 cmHg and 30 °C, the pure Cs membrane had a CO_2_ permeation rate of 2.64 × 10^−5^ cm^3^ (STP) cm^−2^∙s^−1^∙cm∙Hg^−1^ and an ideal CO_2_/N_2_ selectivity of 2.9, while the blinding composite membrane (Cs:SPDMAEMA-8 = 4:4) had a CO_2_ permeation rate of 8.54 × 10^−4^ cm^3^ (STP) cm^−2^∙s^−1^∙cm∙Hg^−1^ and an ideal CO_2_/N_2_ selectivity of 35.2. This may be because star PDMAEMA can offer a larger free volume due to its unique properties compared with their linear analogues. Moreover, increasing the content of star polymer will cause increment of the amount of effective CO_2_ facilitated carriers in the membrane. However, when Cs:SPDMAEMA-8 equals 1:7, the blinding composite membrane had a bad mechanical strength and was easy to break with the increasing pressure so that the blinding composite lost the separation performance.

## 3. Experimental Section

### 3.1. Materials

**Scheme 1:**
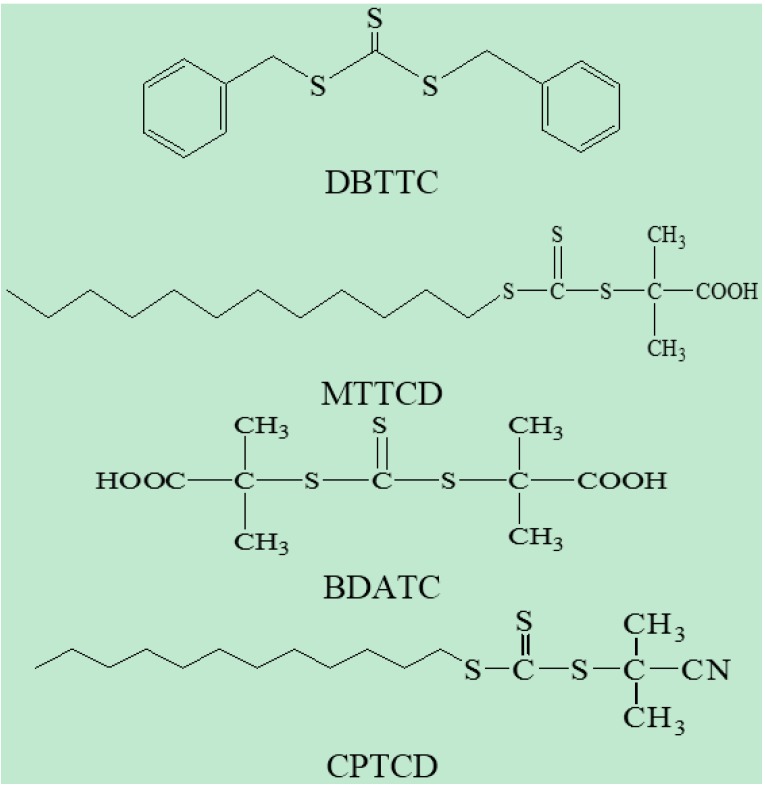
Chemical structures of DBTTC, MTTCD, BDATC and CPTCD.

DMAEMA was purified under reduced pressure to remove the inhibitor before use. DVB (Aladdin chemistry Co., Ltd., Shanghai, China, 80%) was washed with a 5~10 wt % NaOH solution, dried with anhydrous magnesium sulphate for 24~48 h, and then distilled under vacuum. 2,2'-Azo-bis (isobutyronitrile) (AIBN) (Fluka, 98%) was recrystallized from ethanol at 40 °C. Toluene was refluxed over sodium for 24 h, and then distilled prior to use. RAFT agents were prepared according to the method described in documents. DBTTC was synthesized according to the methods reported in the literature [44], IR (KBr): 1453.4, 1064.6. ^1^H NMR (CDCl_3_): 4.67 ppm (s, 4H, 2-CH_2_-), 7.15–7.25 ppm (m, 10H, 2aromatic H). MTTCD was synthesized according to the methods reported in the literature [45], IR (KBr): 1725, 1075. ^1^H NMR (CDCl_3_): 0.99 ppm (t, 3H), 1.37–1.47 ppm (m, 20H), 1.75 ppm (s, 6H), 3.42 ppm (t, 2H). BDATC was synthesized according to the methods reported in the literature [45], IR (KBr, cm^−1^): 1700, 1060. ^1^H NMR (DMSO, δ): 1.59 (s,12H), 12.91 (s, 2H). CPTCD was synthesized according to the methods reported in the literature [46]. IR (KBr): 2234.3, 1075. ^1^H NMR (CDCl_3_): 0.88 ppm (t, 3H), 1.22–1.70 ppm (m, 20H), 1.87 ppm (s, 6H), 3.27 ppm (t, 2H). Their molecular structures were shown in Scheme 1. Cs was purchased from Aladdin Chemistry Co., Ltd., Shanghai, China. The PS UF membrane (*M*_w_ cutoff 30,000) was supplied by Development Center of Water Treatment Technology, Hangzhou, China. All other chemicals used in the experiments were commercially analytical grade.

### 3.2. Polymerization

#### 3.2.1. Preparation of Linear PDMAEMA Macro Transfer Agent

Polymerization was carried out in sealed three-neck flasks. A general synthetic procedure is as follows: DMAEMA, AIBN, DBTTC or MTTCD or BDATC or CPTCD with various molar ratios (listed in Table 1), and toluene (14.0 mL) were added into a 100 mL three-neck flask. After the mixture was bubbled with nitrogen for 30 min, the flask was immersed into an oil bath preheated to 70 °C under N_2_ atmosphere while stirring. After a prescribed time, the reaction was quenched by plunging the flask into ice water to halt the polymerization. The pure polymer was obtained by precipitation of the reaction mixture in petroleum ether three times, collected by filtration, and finally dried in a vacuum oven at 40 °C for 24 h. The monomer conversions were determined gravimetrically.

When the initiator was much less than the CTA, the theoretical molecular weight could be calculated according to the following Equation (1):

(1) $$M_{n,th}=M_{n,Raft}+\frac{{\left[DMAEMA\right]}_{0}\times M\times x}{{\left[RAFT\right]}_{0}}$$

where [*DMAEMA*]_0_ and [*RAFT*]_0_ are the starting concentrations of the DMAEMA and the RAFT agent, respectively. *x* is the conversion, and *M* is the molar mass of the DMAEMA. *M*_n,Raft_ is the molar mass of the RAFT agent. The contribution of the molar mass of the chains initiated by AIBN was neglected.

#### 3.2.2. Preparation of Star Polymers

**Scheme 2:**
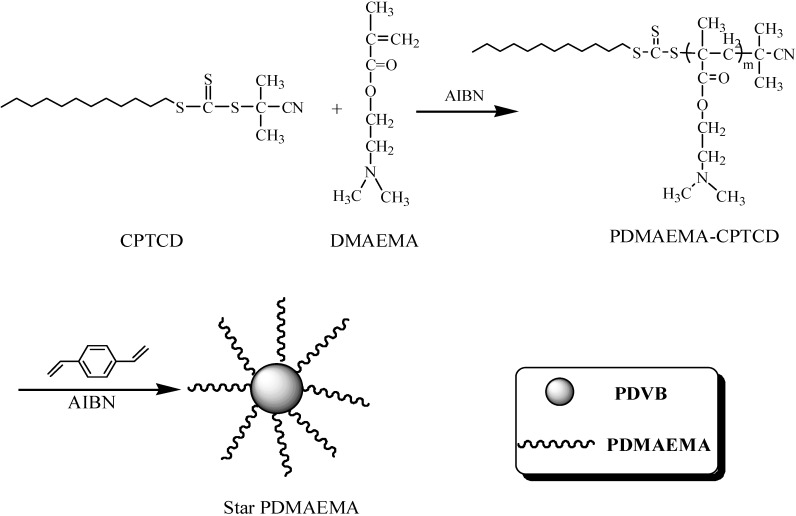
The synthesis route for SPDMAEMA in presence of CPTCD.

The macro RAFT agent, PDMAEMA-CPTCD (Run 9 in Table 1, 0.415 g, *M*_n,GPC_ = 8325 g/mol), AIBN (4.11 mg, 0.025 mmol) and DVB with various concentrations were dissolved in toluene (8.26 mL) in the sealed three-neck flasks. Seven solutions with various molar ratios of DVB:PDMAEMA-CPTCD (25:1, 35:1, 40:1, 45:1, 50:1, 55:1, 60:1) were used for determining optimum preparation conditions of star polymers. After the mixture was bubbled with nitrogen for 30 min, the flask was then subsequently immersed into an oil bath preheated at 100 °C under N_2_ atmosphere with stirring for 12 h. The reaction was stopped by placing the flask into an ice water. The star PDMAEMA (SPDMAEMA) was precipitated from petroleum ether, and dried in a vacuum oven at 40 °C for 24 h. At last, the separation of star polymers from linear polymers contaminant was carried out by the precipitation fractionation technique. Polymer stars using CPTCD as the chain transfer agent were prepared according to Scheme 2.

### 3.3. The Blinding Composite Membranes Preparation

Quantities of 0.1 g Cs and 0.1 g SPDMAEMA-8 were respectively dissolved in 4.9 g, 5.0 wt % HAc aqueous solution to form a 2.0 wt % solution and stirred with a magneton until they were completely dissolved. Cs solution and SPDMAEMA-8 solution of 2.0 wt % were mixed by volume ratios (*v*/*v*), such as 8:0, 7:1, 6:2, 4:4, 2:6, 1:7. Then the solutions were filtered to remove any non-dissolved suspend matters. The blinding composite membranes were prepared by casting the polymer cast solution on the PS ultrafiltration membrane, followed by dried at room temperature for 24 h. Finally, The CO_2_ permeation performance of the prepared membranes was determined under the wet condition.

### 3.4. Measurements and Instruments

The chemical structures of PDMAEMA and star PDMAEMA were studied by FT-IR and ^1^H NMR spectroscopy. FT-IR spectroscopy on a Nicolet 6700 FT-IR spectrophotometer with the polymer samples dispersed in KBr pellets. ^1^H NMR spectra were recorded on a UNITY-plus 400 M nuclear magnetic resonance spectrometer using CDCl_3_ as the solvent and tetramethylsilane (TMS) as internal standard. XRD was used to test the crystallinity of the polymers. The number-average molecular weight (*M*_n_), weight-average molecular weight (*M*_W_), and polydispersity (*M*_W_/*M*_n_) of the polymers were determined by gel permeation chromatograph (GPC) with a set of a Waters 1515 isocratic HPLC pump and a Waters 2414 Refractive index detector under the control of Breeze software run by an external PC. DMF+0.05M LiBr was used as the mobile phase at a flow rate of 1.0 mL/min at 60 °C and monodisperse polymethyl methacrylate (PMMA) standards were used in the calibration of molecular weights.

The permeation testing set-up used were similar to reported previously. The effective membrane area is 19.6 cm^2^. The obtained membrane was tested by feed gas which was pure gas of CO_2_ and N_2_. The permeation rate of the gas was calculated from the flow rate of H_2_ which is the sweep gas and the integral area of the penetrate gas, CO_2_ and N_2_, by the gas chromatograph with a thermal conductivity detector. The permeation rate and the selectivity are given by are given by Ri = Ni/Δpi, S_CO2/N2_ = R_CO2_/R_N2_ where Ni is the permeation flux of permeate gas, Δpi is the trans-membrane partial pressure difference. R_CO2_, R_N2_ are the permeation rate of CO_2_ and N_2_, respectively.

## 4. Conclusions

RAFT polymerization of DMAEMA has been successfully carried out in the presence of DBTTC, MTTCD, BDATC and CPTCD as the chain transfer agents and AIBN as the initiator. The results showed that CPTCD was the effective RAFT agent for the RAFT polymerization of DMAEMA. The polymerization of DMAEMA with CPTCD as the chain transfer agent showed “living”/controlled characteristics and exhibited pseudo-first-order kinetics. Star PDMAEMA has been successfully prepared by the RAFT polymerization of DVB using PDMAEMA-CPTCD as macro RAFT agent and the pure star PDMAEMA was also obtained by the precipitation fractionation technique. We can see from the results that the polydispersity index of SPDMAEMA-8 (PDI = 1.43) was apparently lower than SPDMAEMA-4 (PDI = 2.10, DVB/PDMAEMA-CPTCD = 45). The chemical composition and structure of the linear and star polymers were characterized by GPC, FTIR, ^1^H NMR, XRD analysis.

For the pure Cs membrane, CO_2_ permeation rate and ideal selectivity of the blending composite membrane both have a great improvement with increasing the content of SPDMAEMA-8. When at a feed gas pressure of 37.5 cmHg and 30 °C, the blinding composite membrane (Cs:SPDMAEMA-8 = 4:4) had a CO_2_ permeation rate of 8.54 × 10^−4^ cm^3^ (STP) cm^−2^∙s^−1^∙cm∙Hg^−1^ and a N_2_ permeation rate of 6.76 × 10^−5^cm^3^ (STP) cm^−2^∙s^−1^∙cm∙Hg^−1^, and an ideal CO_2_/N_2_ selectivity of 35.2.
